# Supplementary material for: Cryo-EM structures of HKU2 and SADS-CoV spike glycoproteins provide insights into coronavirus evolution
Source: Nat Commun. 2020 Jun 17;11:3070. doi: 10.1038/s41467-020-16876-4 (PMC7300015; doi:10.1038/s41467-020-16876-4)
Supplement: Supplementary file 1 — Supplementary Information [file 41467_2020_16876_MOESM1_ESM.pdf]

Cryo-EM structures of HKU2 and SADS-CoV spike glycoproteins provide insights into coronavirus evolution

Jinfang Yu[1], Shuyuan Qiao[1], Runyu Guo[1], Xinquan Wang[1,*]

[1]The Ministry of Education Key Laboratory of Protein Science, Beijing Advanced Innovation Center for Structural Biology, Beijing Frontier Research Center for Biological Structure, Collaborative Innovation Center for Biotherapy, School of Life Sciences, Tsinghua University, 100084 Beijing, China

[*]To whom correspondence should be addressed.

The authors declare no competing financial interests. Correspondence and requests for materials should be addressed to X.W. (xinquanwang@tsinghua.edu.cn).

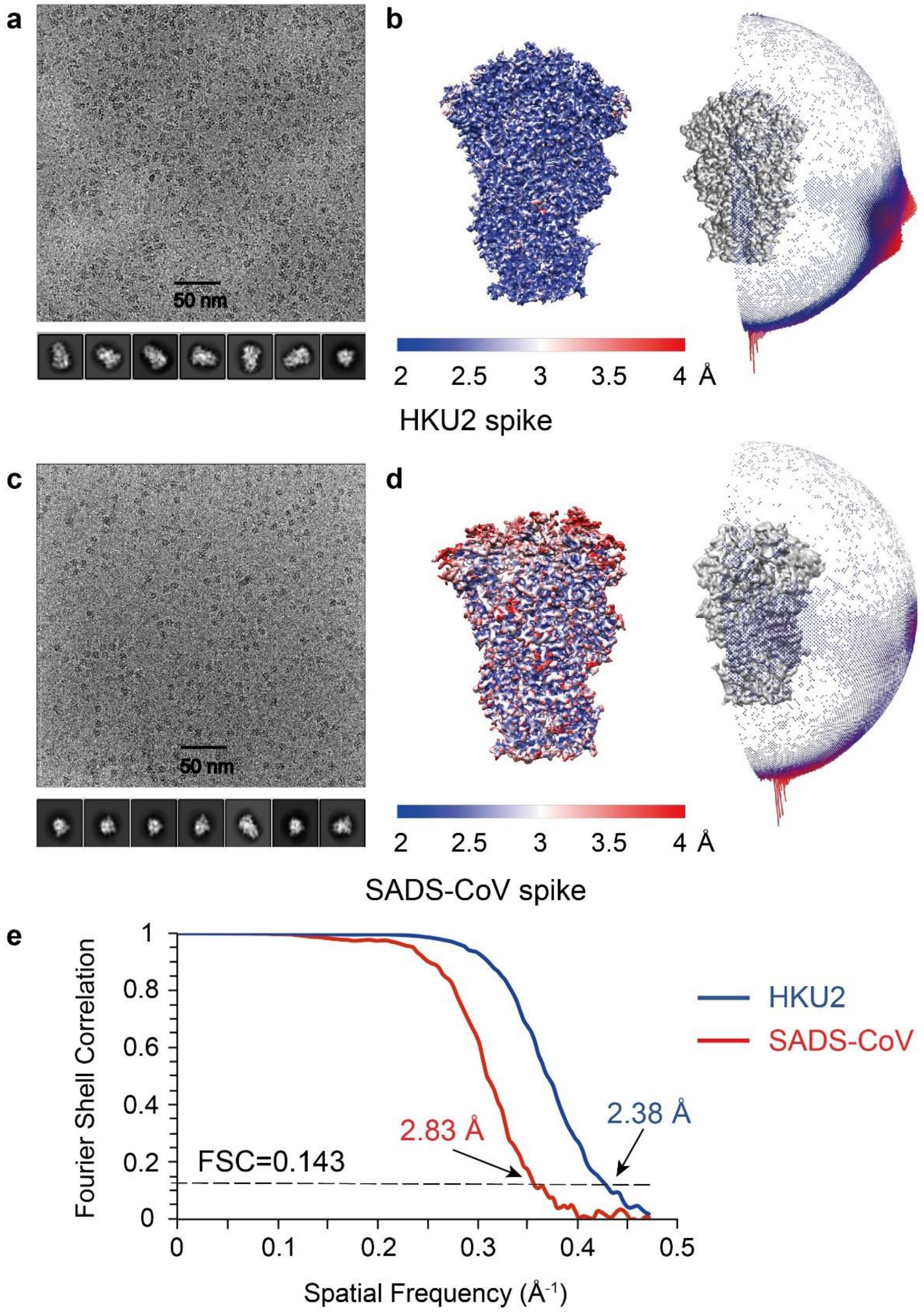

Supplementary Figure 1. Cryo-EM data and the statistics of the final density maps. (a) Representative cryo-EM micrograph and selected 2D class averages of HKU2 spike. We have collected 7663 micrographs. **(b)** Local resolution (Å) plotted on the cryo-EM map (left panel) as a heat map and particle orientation distribution (right panel) of HKU2 spike. The cryo-EM map is contoured at 3 RMS. The color scale from 2 Å to 4 Å is shown at the bottom of the local resolution map. The red cylinders in the right panel represent the particles on these orientations; heights of cylinders represent the relative numbers of particles. **(c)** Representative cryo-EM micrograph and selected 2D class averages of SADS-CoV spike. We have collected 4569 micrographs. **(d)** Local resolution (Å) plotted on the cryo-EM map (left panel) as a heat map and particle orientation distribution (right panel) of SADS-CoV spike. The cryo-EM map is contoured at 4 RMS. The color scale from 2 Å to 4 Å is shown at the bottom of the local resolution map. The red cylinders in the right panel represent the particles on these orientations; heights of cylinders represent the relative numbers of particles. **(e)** Gold-standard Fourier Shell Correlation (FSC) curves of the final density maps. The final resolution of HKU2 spike is 2.38 Å; the final resolution of SADS-CoV spike is 2.83 Å.

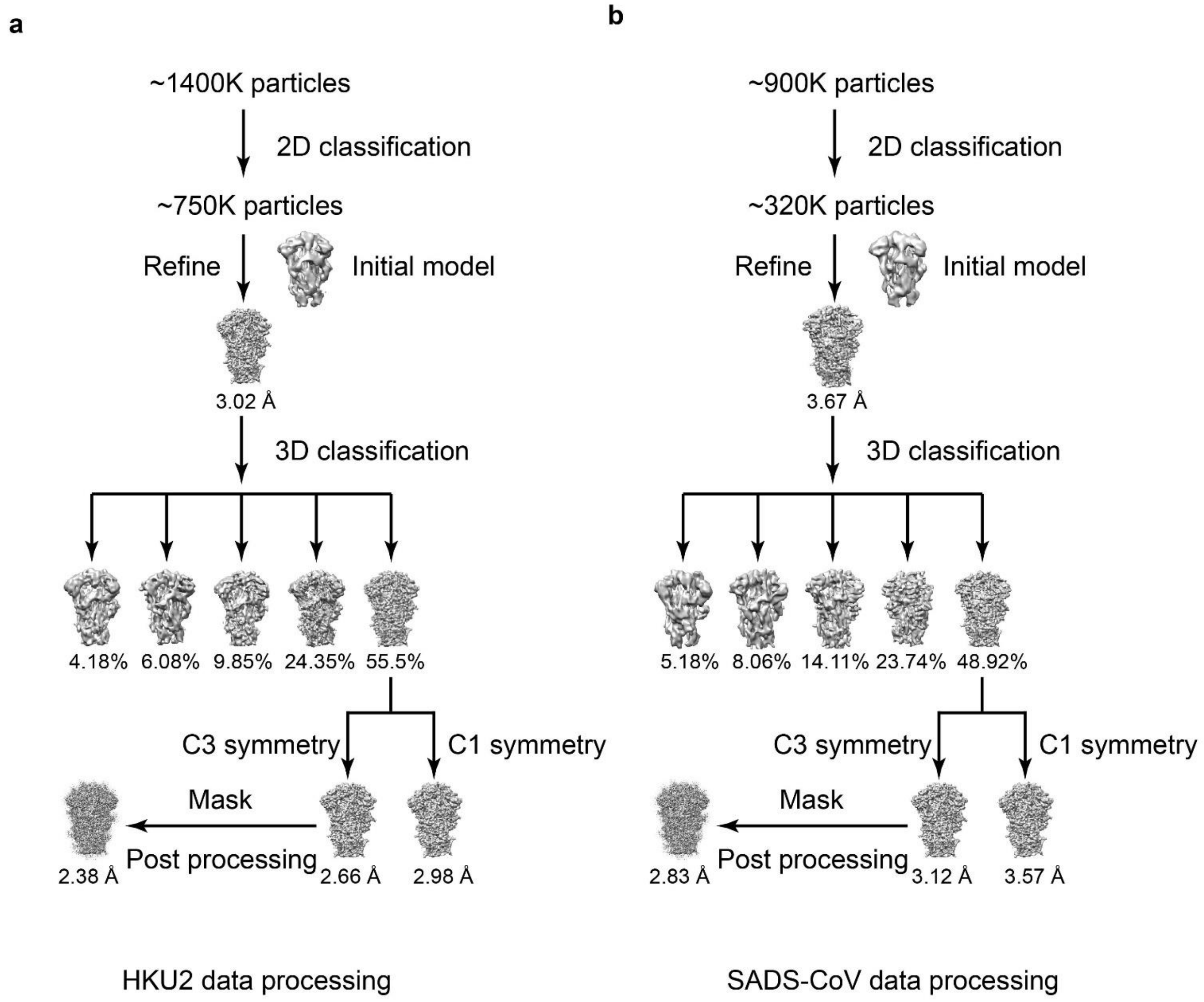

Supplementary Figure 2. 3D reconstruction workflow. (a) 3D reconstruction workflow of HKU2 spike cryo-EM data. **(b)** 3D reconstruction workflow of SADS-CoV spike cryo-EM data.

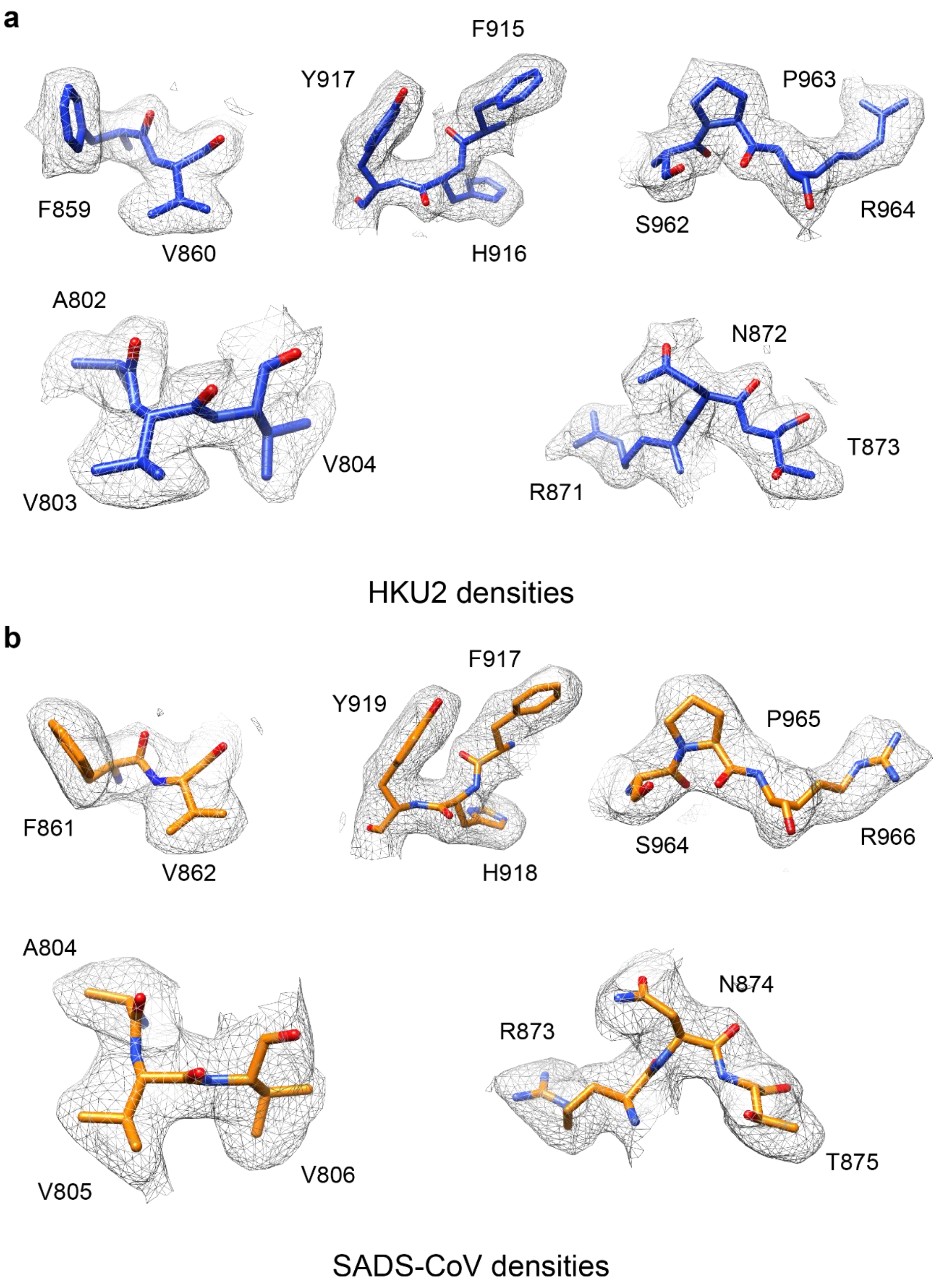

Supplementary Figure 3. The representative density maps of HKU2 and SADS-CoV. (a) The representative density maps of HKU2 spike. The map is contoured at 2.6 RMS to show the density. **(b)** The representative density maps of SADS-CoV spike. The map is contoured at 3 RMS to show the density.

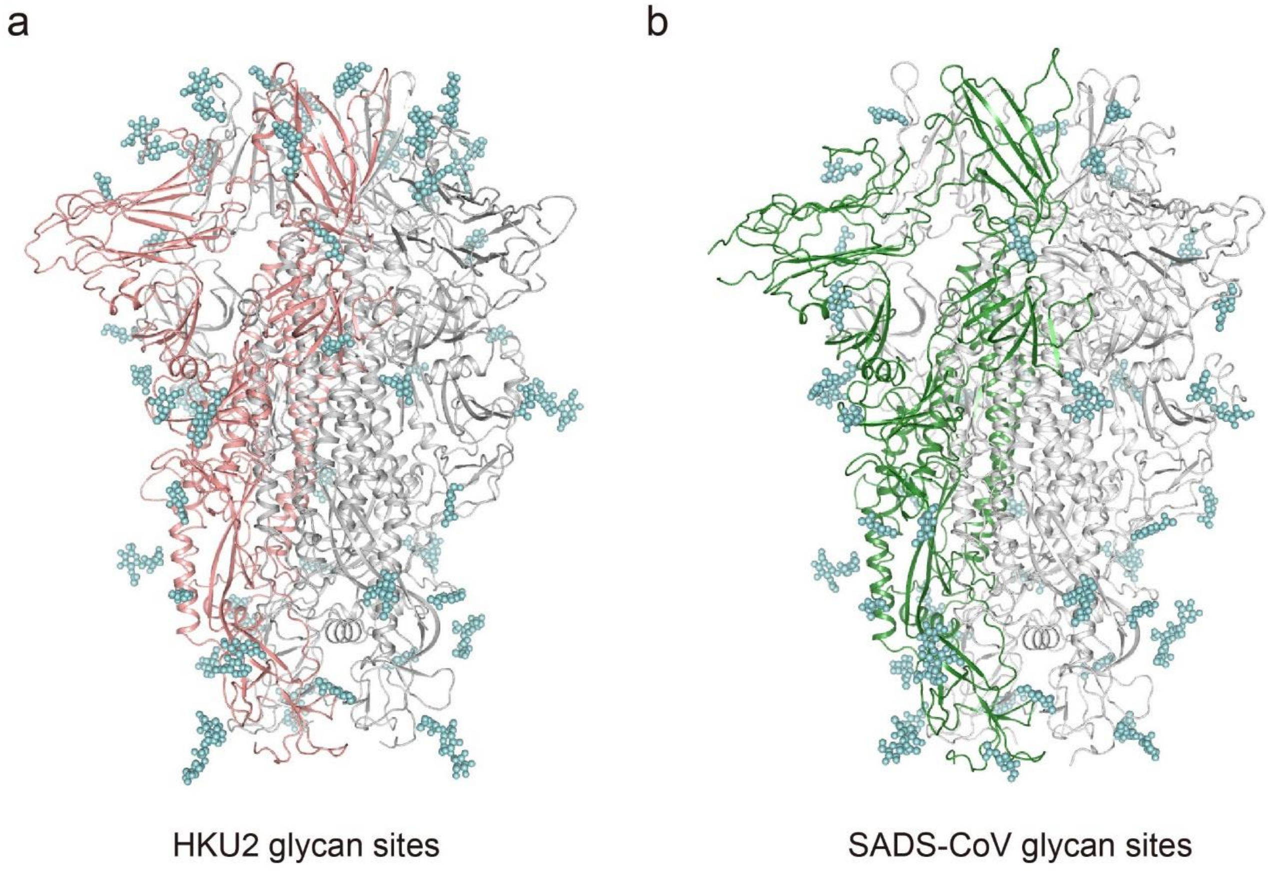

Supplementary Figure 4. Glycan sites of HKU2 spike and SADS-CoV spike. (a) Glycan sites of HKU2 spike. Glycans are colored cyan. One monomer of HKU2 spike is colored salmon. The others are colored gray. **(b)** Glycan sites of SADS-CoV spike. Glycans are colored cyan. One monomer of SADS-CoV spike is colored green. The others are colored gray.

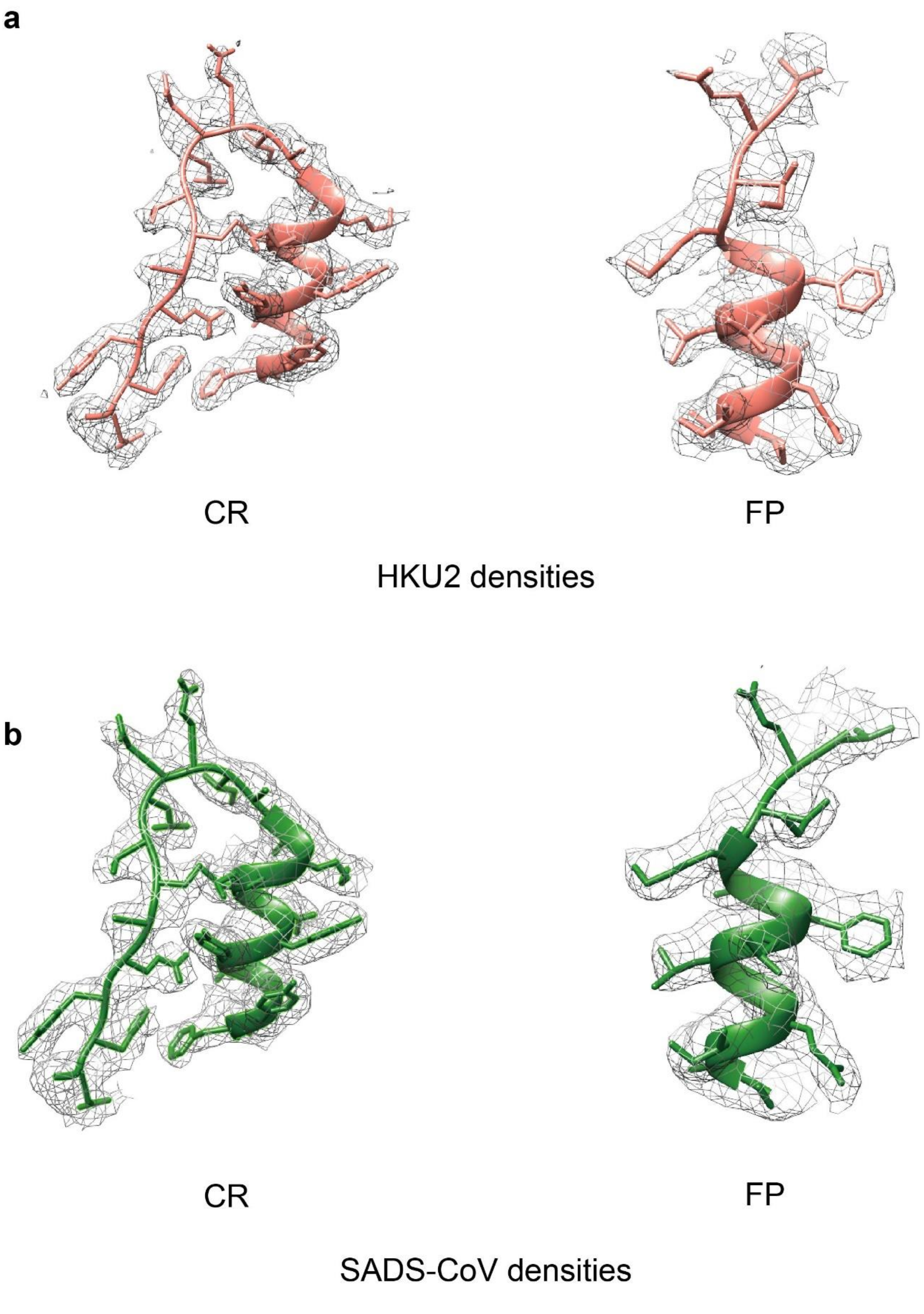

Supplementary Figure 5. The density maps of CR and FP. (a) The CR and FP density maps of HKU2 spike. The map is contoured at 2.6 RMS to show the density. CR, connecting region. FP, fusion peptide. **(b)** The CR and FP density maps of SADS-CoV spike. The map is contoured at 3 RMS to show the density. CR, connecting region. FP, fusion peptide.

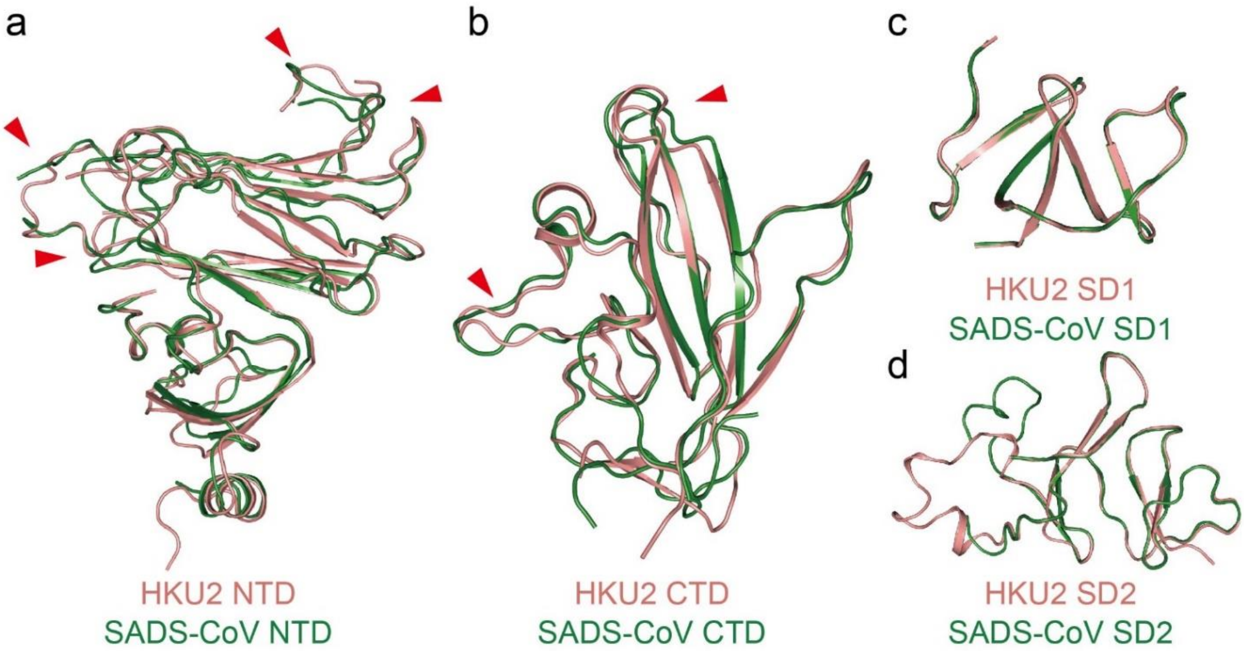

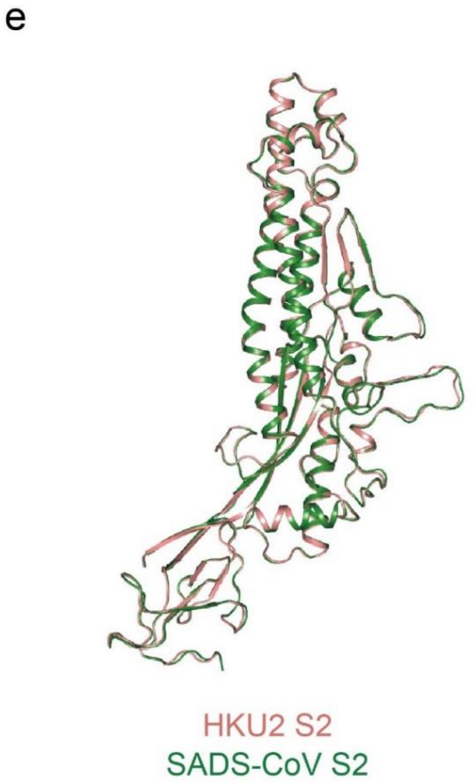

f

HKU2&SADS-CoV comparison

	Sequence similarity	Structure similarity (RMSD, Å)
NTD	70%	1.2
CTD	82%	1.1
SD1	85%	0.36
SD2	84%	0.27
S2	95%	0.29
Full length	86%	0.53

Supplementary Figure 6. Structural comparison of HKU2 monomer and SADS-CoV monomer. (a) Structural comparison of HKU2 NTD and SADS-CoV NTD. The variations of loops are indicated by red triangles. HKU2 NTD is colored salmon; SADS-CoV NTD is colored green. **(b)** Structural comparison of HKU2 CTD and SADS-CoV CTD. The variations of loops are indicated by red triangles. The colors are the same as in a. **(c)** Structural comparison of HKU2 SD1 and SADS-CoV SD1. The colors are the same as in a. **(d)** Structural comparison of HKU2 SD2 and SADS-CoV SD2. The colors are the same as in a. **(e)** Structural comparison of HKU2 S2 and SADS-CoV S2. The colors are the same as in a. **(f)** Quantitative comparison between segments of HKU2 spike and SADS-CoV spike. The RMSD value is calculated using PyMOL.

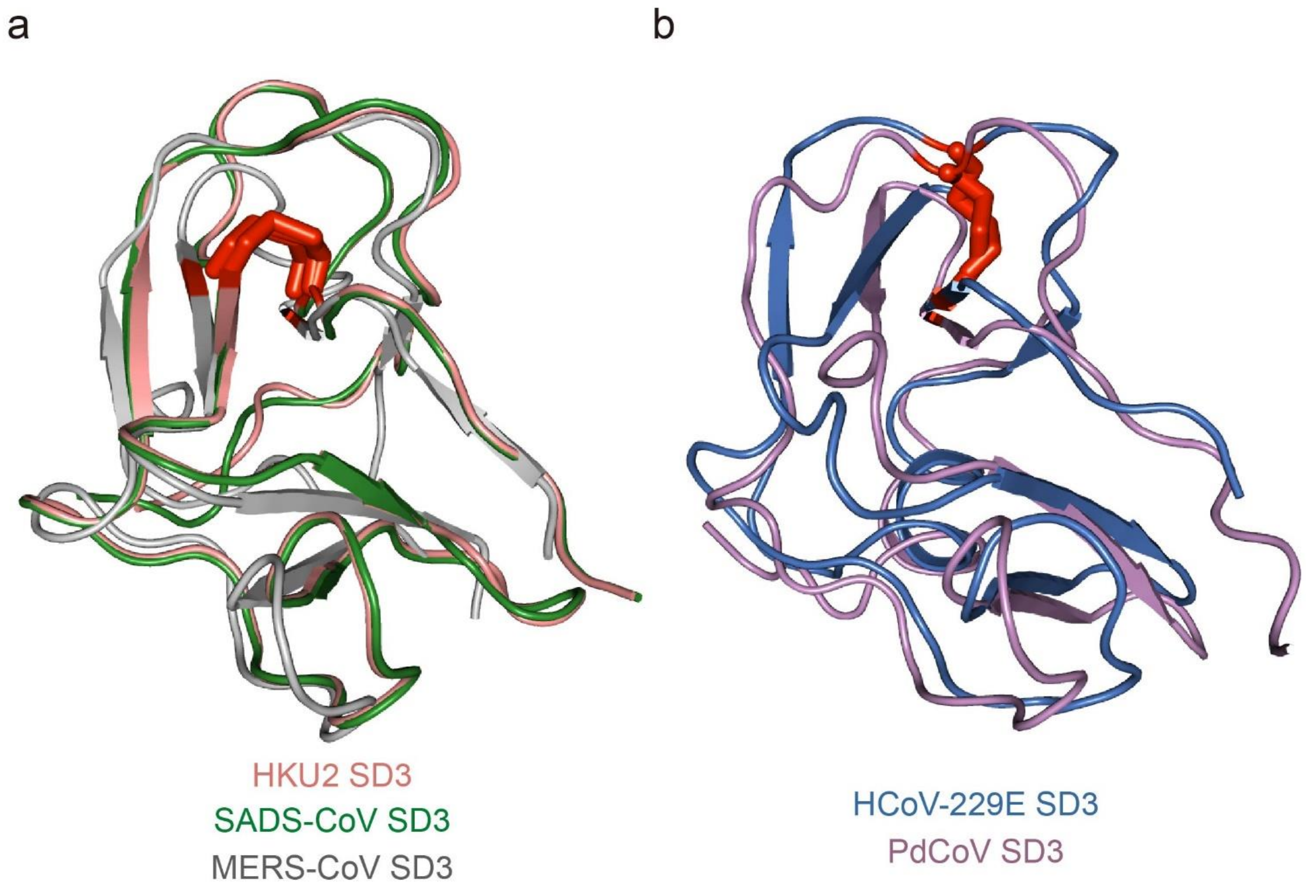

Supplementary Figure 7. Two types of disulfide bonds in SD3. (a) The disulfide bond position in SD3 is the same in HKU2, SADS-CoV and MERS-CoV spikes. HKU2 SD3 is colored salmon; SADS-CoV SD3 is colored green; MERS-CoV SD3 is colored gray. PDB code: MERS-CoV, 6Q05. **(b)** The disulfide bond position in SD3 is the same in other coronavirus spikes, which are represented by HCoV-229E and PdCoV. HCoV-229E SD3 is colored marine; PdCoV SD3 is colored violet. PDB codes: HCoV-229E, 6U7H; PdCoV, 6B7N.

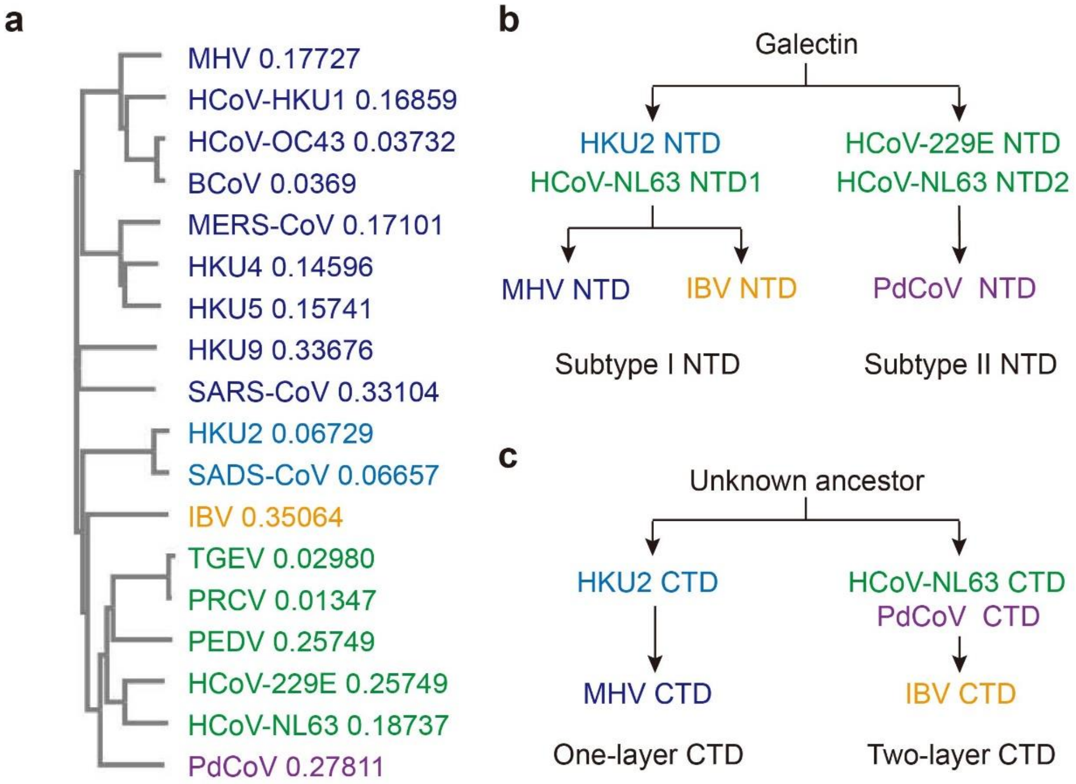

d

RMSD(Å)	HCoV-229E	HCoV-NL63	PEDV	TGEV	PRCV	BCoV	HCoV-HKU1	MHV	MERS-CoV	SARS-CoV	HCoV-OC43	IBV	PdCoV
HKU2-NTD	4	2.7(I) 4.3(II)	2.3(I) 4.1(II)	NA	NA	3	3	2.9	3.2	3.1	2.8	3.6	3.9
SADS-NTD	4	3.1(I) 4.1(II)	2.6(I) 4.1(II)	NA	NA	3	3	2.9	3.5	3.5	3.3	3.5	3.8
HKU2-CTD	4.1	3.9	3.9	4.7	4.6	NA	3.4	3	3	3.4	3	3.5	4.1
SADS-CTD	4	3.6	3.5	4.4	3.9	NA	3.1	3.2	3.1	3.6	3.2	3.6	3.7
HKU2-SD1	1.7	2	1.8	NA	NA	NA	1.7	2.1	1.9	1.7	1.6	1.7	2
SADS-SD1	1.8	2.2	2	NA	NA	NA	1.7	2.1	1.9	1.7	1.6	1.8	2.1
HKU2-SD2	2.8	2.4	2.8	NA	NA	NA	2.3	3.1	2.1	3.1	2	2.1	2.9
SADS-SD2	2.8	2.7	2.9	NA	NA	NA	2.4	2.4	2	2.3	2.2	2.5	2.7
HKU2-S2	4.6	3.5	3.6	NA	NA	NA	3	2.9	2.6	3.4	3.5	3.7	3.6
SADS-S2	4.6	3.5	3.7	NA	NA	NA	3	2.9	2.7	3.5	3.5	3.7	3.7

Supplementary Figure 8. Evolution relationship of coronaviruses. (a) Phylogenetic tree of coronaviruses. β-CoVs are colored dark blue. HKU2 and SADS-CoV are colored light blue. γ-CoV (IBV) is colored orange. α-CoVs are colored green. δ-CoV (PdCoV) is colored violet. The relative distances are calculated on Clustal Omega. **(b)** Putative evolution pathways of coronavirus NTDs. The colors are the same as in a. **(c)** Putative evolution pathways of coronavirus CTDs. The colors are the same as in a. **(d)** Quantative comparison between segments of HKU2 (SADS-CoV) and coronaviruses from different genera. The colors are the same as in a. The RMSD values are calculated on DALI server.

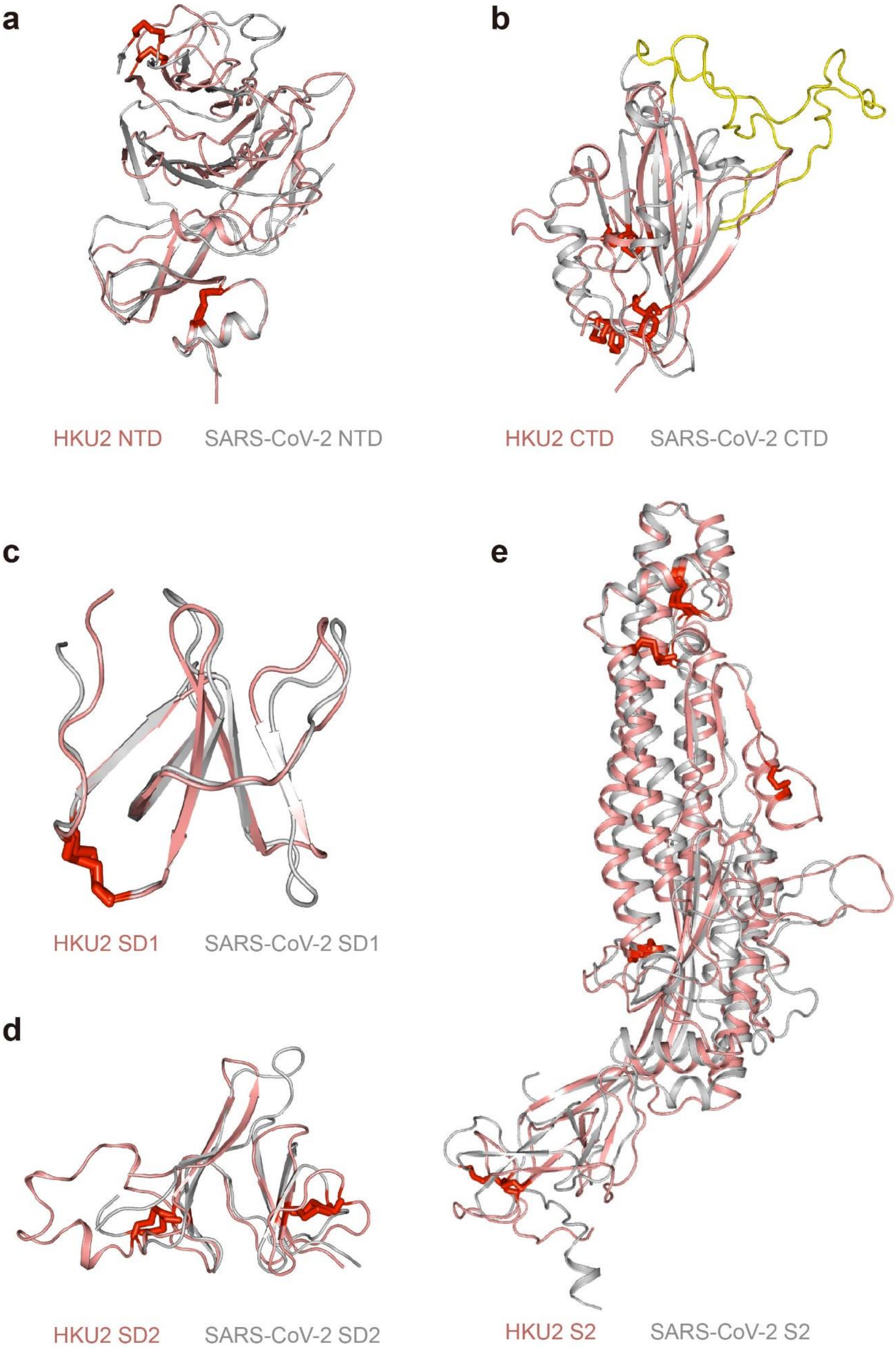

Supplementary Figure 9. Structural alignment of each subunit of SARS-CoV-2 and HKU2. (a) Structural comparison of HKU2 NTD and SARS-CoV-2 NTD. HKU2 NTD is colored salmon; SARS-CoV-2 NTD is colored gray. **(b)** Structural comparison of HKU2 CTD and SARS-CoV-2 CTD. Extra domain of SARS-CoV-2 CTD is colored yellow. The colors are the same as in a. **(c)** Structural comparison of HKU2 SD1 and SARS-CoV-2 SD1. The colors are the same as in a. **(d)** Structural comparison of HKU2 SD2 and SARS-CoV-2 SD2. The colors are the same as in a. **(e)** Structural comparison of HKU2 S2 and SARS-CoV-2 S2. Conserved disulfide bonds are shown as red sticks. PDB codes for SARS-CoV-2: 6VXX and 6M0J.
