# Supplementary material for: Cryo-EM structures of HKU2 and SADS-CoV spike glycoproteins provide insights into coronavirus evolution
Source: Nat Commun. 2020 Jun 17;11:3070. doi: 10.1038/s41467-020-16876-4 (PMC7300015; doi:10.1038/s41467-020-16876-4)
Supplement: Supplementary file 3 — Reporting Summary [file 41467_2020_16876_MOESM3_ESM.pdf]

nature research

Corresponding author(s): Xinquan Wang

Last updated by author(s): May 18, 2020

Reporting Summary

Nature Research wishes to improve the reproducibility of the work that we publish. This form provides structure for consistency and transparency in reporting. For further information on Nature Research policies, seeAuthors & Referees and theEditorial Policy Checklist .

Statistics

For all statistical analyses, confirm that the following items are present in the figure legend, table legend, main text, or Methods section.

n/a	Confirmed	
☒	☐	The exact sample size (n) for each experimental group/condition, given as a discrete number and unit of measurement
☒	☐	A statement on whether measurements were taken from distinct samples or whether the same sample was measured repeatedly
☒	☐	The statistical test(s) used AND whether they are one- or two-sided<br>*Only common tests should be described solely by name; describe more complex techniques in the Methods section.*
☒	☐	A description of all covariates tested
☒	☐	A description of any assumptions or corrections, such as tests of normality and adjustment for multiple comparisons
☒	☐	A full description of the statistical parameters including central tendency (e.g. means) or other basic estimates (e.g. regression coefficient) AND variation (e.g. standard deviation) or associated estimates of uncertainty (e.g. confidence intervals)
☒	☐	For null hypothesis testing, the test statistic (e.g. F, t, r) with confidence intervals, effect sizes, degrees of freedom and P value noted<br>*Give P values as exact values whenever suitable.*
☒	☐	For Bayesian analysis, information on the choice of priors and Markov chain Monte Carlo settings
☒	☐	For hierarchical and complex designs, identification of the appropriate level for tests and full reporting of outcomes
☒	☐	Estimates of effect sizes (e.g. Cohen's d, Pearson's r), indicating how they were calculated

Our web collection on statistics for biologists contains articles on many of the points above.

Software and code

Policy information about availability of computer code

Data collection	There is no open source software for collecting data.
Data analysis	The AutoEmation is a collecting software but is not open source.RELION3.0, Resmap1.1.4, COOT 0.8.6 and PHENIX 1.15.2-3472 were used at the determination of the structure for data processing , model building and refinement. PyMOL 1.8.x and chimera 1.13 was used to generate the structural figures.

For manuscripts utilizing custom algorithms or software that are central to the research but not yet described in published literature, software must be made available to editors/reviewers. We strongly encourage code deposition in a community repository (e.g. GitHub). See the Nature Research guidelines for submitting code & software for further information.

Data

Policy information about availability of data

All manuscripts must include a data availability statement. This statement should provide the following information, where applicable:

- Accession codes, unique identifiers, or web links for publicly available datasets
- A list of figures that have associated raw data
- A description of any restrictions on data availability

NCBI was used for downloading the published HKU2 and SADS-CoV sequences to do the research. Cryo-EM structure presented in this work have been deposited in the Protein Data Bank (PDB) under the accession codes 6M15 and 6M16. The map have been deposited into the EMDB under codes EMD-30037and EMD-30038.

Field-specific reporting

Please select the one below that is the best fit for your research. If you are not sure, read the appropriate sections before making your selection.

Life sciences study design

All studies must disclose on these points even when the disclosure is negative.

Sample size	Two separate proteins were purified
Data exclusions	No data excluded.
Replication	These two proteins were stably expressed
Randomization	These two proteins were stably expressed
Blinding	These two proteins were stably expressed

Reporting for specific materials, systems and methods

We require information from authors about some types of materials, experimental systems and methods used in many studies. Here, indicate whether each material, system or method listed is relevant to your study. If you are not sure if a list item applies to your research, read the appropriate section before selecting a response.

Materials & experimental systems

n/a	Involved in the study	
☒	☐	Antibodies
☐	☒	Eukaryotic cell lines
☒	☐	Palaeontology
☒	☐	Animals and other organisms
☒	☐	Human research participants
☒	☐	Clinical data

Methods

n/a	Involved in the study	
☒	☐	ChIP-seq
☒	☐	Flow cytometry
☒	☐	MRI-based neuroimaging

Eukaryotic cell lines

Policy information about cell lines

Cell line source(s)	SF9 and Hi5 cells were bought from ATCC
Authentication	SF9：refered to website: https://www.atcc.org/products/all/CRL-1711.aspx<br>Hi5：refered to website<br>These two cell lines are all available in commercial company.
Mycoplasma contamination	We confirm that all cell lines were negative for mycoplasma contamination
Commonly misidentified lines (See ICLAC register)	No commonly misidentified cell lines were used
